# The subcommissural organ maintains features of neuroepithelial cells in the adult mouse

**DOI:** 10.1111/joa.13709

**Published:** 2022-05-31

**Authors:** Laarni Grace Corales, Hitoshi Inada, Kotaro Hiraoka, Shun Araki, Shinya Yamanaka, Takako Kikkawa, Noriko Osumi

**Affiliations:** ^1^ Department of Developmental Neuroscience Tohoku University Graduate School of Medicine Sendai Japan; ^2^ Laboratory of Health and Sports Sciences, Division of Biomedical Engineering for Health and Welfare Tohoku University Graduate School of Biomedical Engineering Sendai Japan; ^3^ Division of Cyclotron Nuclear Medicine, Cyclotron and Radioisotope Center Tohoku University Sendai Japan

**Keywords:** neural stem/progenitor cells, Pax6, subcommissural organ (SCO)

## Abstract

The subcommissural organ (SCO) is a part of the circumventricular organs located in the dorsocaudal region of the third ventricle at the entrance of the aqueduct of Sylvius. The SCO comprises epithelial cells and produces high molecular weight glycoproteins, which are secreted into the third ventricle and become part of Reissner's fibre in the cerebrospinal fluid. Abnormal development of the SCO has been linked with congenital hydrocephalus, a condition characterized by excessive accumulation of cerebrospinal fluid in the brain. In the present study, we characterized the SCO cells in the adult mouse brain to gain insights into the possible role of this brain region. Immunohistochemical analyses revealed that expression of Pax6, a transcription factor essential for SCO differentiation during embryogenesis, is maintained in the SCO at postnatal stages from P0 to P84. SCO cells in the adult brain expressed known neural stem/progenitor cell (NSPC) markers, Sox2 and vimentin. The adult SCO cells also expressed proliferating marker PCNA, although expression of another proliferation marker Ki67, indicating a G_2_/M phase, was not detected. The SCO cells did not incorporate BrdU, a marker for DNA synthesis in the S phase. Therefore, the SCO cells have a potential for proliferation but are quiescent for cell division in the adult. The SCO cells also expressed GFAP, a marker for astrocytes or NSPCs, but not NeuN (for neurons). A few cells positive for Iba1 (microglia), Olig2 (for oligodendrocytes) and PDGFRα (oligodendrocyte progenitors) existed within or on the periphery of the SCO. These findings revealed that the SCO cells have a unique feature as secretory yet immature neuroepithelial cells in the adult mouse brain.

## Graphical abstract

We show that the subcommissural organ maintains features of neuroepithelial cells in the adult mouse. The subcommissural organ expressed Pax6 and two other neural stem cell markers, Sox2 and vimentin, and a proliferation marker PCNA, suggesting proliferating capability. Our results suggest that the SCO has similar features of quiescent neural stem cells in the adult mouse brain.

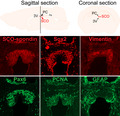

## 1 INTRODUCTION

The subcommissural organ (SCO) is a gland that is part of the circumventricular system within the brain. The SCO is located dorsocaudal of the third ventricle, at the entrance of the aqueduct of Sylvius (Rodriguez et al., 1998), and is known to release high molecular weight glycoproteins that are secreted into the third ventricle and become part of the Reissner's fibre (RF) in the cerebrospinal fluid (Nualart et al., 1991). SCO secretes not only SCO‐spondin but also other proteins such as RF‐glycoproteins (Nualart et al., 1991), transthyretin (Montecinos et al., 2005), and basic fibroblast growth factor (Cuevas et al., 1996). The SCO is a conserved brain gland during chordate evolution, present in lancelet to primates (Guerra et al., 2015), although its function remains largely unknown.

The SCO is one of the first structures to differentiate during early brain development. It is derived from neuroepithelial cells that line the lumen of the diencephalic region of most vertebrates (Huh et al., 2009; Viehweg & Naumann, 1996). In mice, the SCO starts to develop at embryonic day 12.5 (E12.5); the dorsal neuroepithelium differentiates into elongated cells with basal processes that contact the meninges at this stage. The SCO subsequently forms a slight ventricular curve at E14.5, when the SCO cells have visible apical cytoplasm and are immunoreactive to antibodies against Reissner's fibre (Estivill‐Torrus et al., 2001). The SCO is well developed and shows strong immunoreactivity to Reissner's fibre at E16.5 (Estivill‐Torrus et al., 2001). Several transcription factors regulate the development of the SCO in the mouse embryo as shown by mutant mouse models, including Msx1 (Bach et al., 2003; Fernandez‐Llebrez et al., 2004; Ramos et al., 2004), Rfx3 (Baas et al., 2006), Rfx4_v3 (Blackshear et al., 2003) and Pax6 (Estivill‐Torrus et al., 2001). Pax6 transcription factor is well known to be important for the developing central nervous system in multiple aspects by regulating the expression of various target genes (Inada et al., 2018; Kikkawa et al., 2013; Kikkawa et al., 2019; Osumi, 2001; Osumi et al., 2008; Shimoda et al., 2002; Shinohara et al., 2013; Tamai et al., 2007). In the homozygous *Pax6* mutant mouse (*Sey/Sey*), the neuroepithelial cells in the dorsal pretectal midline do not differentiate into glycoprotein secreting SCO cells in both structural and functional aspects, suggesting a critical role of Pax6 for SCO differentiation (Estivill‐Torrus et al., 2001).

Compared with the knowledge about the development of the SCO at the embryonic stage in the mouse, there are limited studies about the SCO in the adult mouse brain. In addition, the exact function of the SCO is still unknown, although it has been suggested that, based on its location, the SCO plays roles in osmoregulation, maintenance of cerebrospinal fluid flow and regulation of the neurogenic niche (Guerra et al., 2015; Perez‐Figares et al., 2001; Rodriguez et al., 1998). In this study, we characterized the SCO organ using several cell type‐specific markers and found that Pax6 expression is maintained in the SCO region of the adult mouse brain. The SCO cells also expressed two other neural stem cell (NSC) markers, Sox2 and vimentin, and a proliferation marker PCNA, suggesting proliferating capability, although they were unlabelled by BrdU assay. Our results suggest that the SCO has similar features of quiescent NSCs in the adult mouse brain.

## 2 METHODS

### 2.1 Animals

C57BL/6J mice used in the study were maintained in the Animal Facility of Tohoku University School of Medicine. All animals were housed in standard cages in a temperature‐ and humidity‐controlled room with a 12‐h light/dark cycle (light on at 8:00 a.m. and off at 8:00 p.m.) and had free access to standard food and water. All experimental procedures were approved by the Ethics Committee for Animal Experiments of Tohoku University Graduate School of Medicine (approval number 2019MdA‐080). The National Institutes of Health guidelines for the care and use of laboratory animals were followed.

### 2.2 Immunohistochemistry

Procedures followed previous literature (Matsumata et al., 2012; Matsumoto et al., 2011; Sakayori et al., 2016). Briefly, C57BL/6J mice from postnatal day 0 (P0) to 3 months old were deeply anaesthetized with isoflurane and perfused intracardially with 4% paraformaldehyde (PFA) in phosphate‐buffered saline (PBS). Brains were then dissected and fixed in 4% PFA overnight at 4°C or immediately proceeded to the sucrose‐soaking step (for vimentin and PDGFRα immunostaining). Brain samples were soaked in PBS containing 20% sucrose overnight at 4°C and frozen in the OCT compound (Sakura Finetek). Cryosections were prepared at 12‐μm thickness using a cryostat (Leica).

Frozen sections were first washed in the Tris Buffered Saline containing 0.1% Tween 20 (TBS‐T) to remove excess OCT compound. Antigen retrieval was performed using 0.01 M citric acid. Blocking was performed by incubating 3% bovine serum albumin (BSA) in TBS‐T for 1 h at room temperature. Sections were incubated with the primary antibodies against the following proteins: goat anti‐SCO‐spondin (1:50; Santa Cruz Biotechnology; sc‐163,332), rabbit anti‐SCO‐spondin (1:200; Biorbyt; ORB507583), Pax6 (1:500; MBL; PD022), Sox2 (1:200; R&D Systems; AF2018), vimentin (1:50; Dako Cytomation; M7020), PCNA (1: 300; Abcam; ab29), Ki67 (1:1000; Abcam; ab15580), GFAP (1:250; Abcam; ab7620), NeuN (1:100; Merck Millipore; MAB377), Iba1 (1:400; WAKO; 019–19,741), Olig2 (1:400; IBL; 18,953) and PDGFRα (1:1000; BD Pharmigen; 558,774) overnight at 4°C. After washing with TBS‐T, the sections were incubated with the corresponding secondary antibodies for 40 min at room temperature with counterstaining by 4′,6‐diamino‐2‐phenylindole (DAPI). Fluorescence images were obtained using a fluorescent microscope (BZ‐X710 KEYENCE, Japan or ZEISS LSM800).

### 2.3 Detection of proliferating cells by 5‐bromo‐2′‐deoxyuridine (BrdU) labelling

Detection of proliferating cells was performed following the methodology of Lee et al. (2012) with modifications. Adult (P75) male mice were injected peritoneally with BrdU (50 mg/ml) for 9 days, sacrificed the next day of the last injection, and immediately perfused. Subsequently, brain samples were processed for cryosectioning as described above. Coronal sections of 12‐μm thickness spanning the entire rostro‐caudal extent of the SCO were prepared. The frozen sections were washed in (TBS‐T) to remove excess OCT compound. Antigen retrieval was performed using 2 M HCl at 37°C for 10 min. Blocking was performed by incubation with 3% BSA in TBS‐T for 1 h at room temperature. Sections were incubated with a primary antibody against BrdU (1:100; AbD Serotec) overnight at 4°C. After washing with TBS‐T, the sections were incubated with a corresponding secondary antibody for 40 min at room temperature with counterstaining by 4′,6‐diamino‐2‐phenylindole (DAPI). Fluorescence images were obtained using a confocal microscope (ZEISS LSM800).

## 3 RESULTS

### 3.1 Pax6 expression is maintained in the SCO at the postnatal stages

**FIGURE 1:**
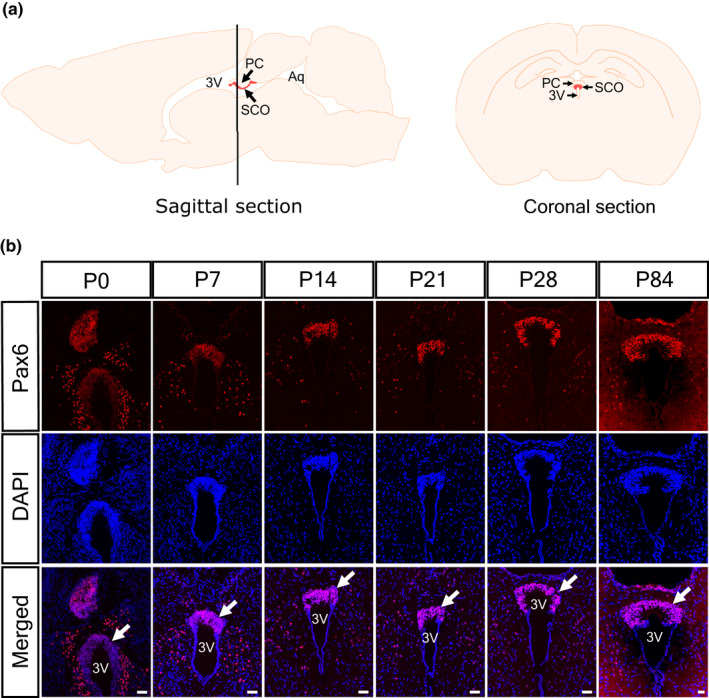
Pax6 expression in the SCO and surrounding area at different postnatal stages. (a) Diagram of the sagittal and coronal section of a postnatal mouse brain showing the location of the SCO (red) relative to the posterior commissure and the third ventricle. (b) Strong Pax6 expression is observed in the SCO (arrows) and ventricular zone at P0 (red). Pax6 expression becomes restricted in the SCO at later stages (P7 to P28) and is still evident at P84. Nuclei are counterstained with DAPI (blue). Arrow indicates the SCO. 3V: Third ventricle, Aq: Sylvius aqueduct, PC: Posterior commissure. Scale bars: 50 μm.

A previous study has reported Pax6 expression in the SCO at E18.5 (Estivill‐Torrus et al., 2001). We first confirmed whether the Pax6 expression was maintained at different postnatal stages from P0 to P84. We morphologically identified the SCO as the U‐shape like structure below the posterior commissure and above the third ventricle in the postnatal brain (Figure 1a). Consistent with the study of Estivill‐Torrus et al. (2001), strong Pax6 expression was observed in the SCO and subventricular zone (SVZ) at P0 (Figure 1b). Pax6 expression in the SCO was relatively weaker than in the SVZ at P0 but became restricted and maintained strongly in the SCO at later stages (P7 to P28). Pax6 continued to be expressed in all the cells in the SCO region of adult mice at P84 (Figure 1b). Therefore, it is suggested that Pax6 could be necessary during the maturation of the SCO.

**FIGURE 2:**
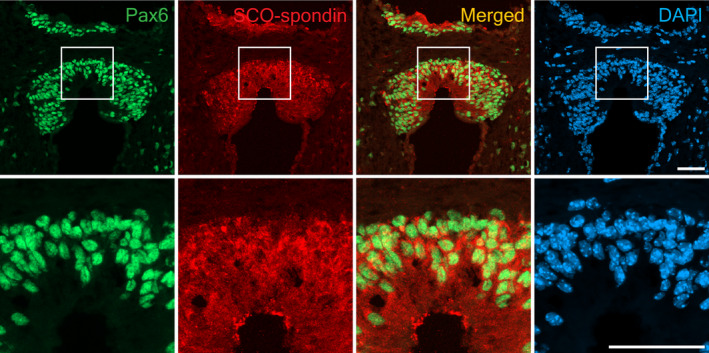
Pax6 expression in the SCO region of a three‐month‐old adult mouse brain fixed with 4% PFA overnight and exposed to antigen retrieval by boiling in the citric acid solution for 12 min. Pax6 (green) and SCO‐specific marker SCO‐spondin (red) expressions are both detected in the region corresponding to the SCO. Nuclei are counterstained with DAPI (blue). Merge images show red and green channels only. Higher magnification shows Pax6 expression in the nucleus and SCO‐spondin expression in the cytoplasm of SCO‐cells. Scale bar: 50 μm.

The specificity of the expression of Pax6 in the SCO was confirmed through double‐immunostaining using antibodies against Pax6 and SCO‐spondin, an SCO specific marker that is a highly expressed glycoprotein in the cytoplasm of ependymal and hypendymal cells of the SCO (Gobron et al., 1996). SCO‐spondin expression was observed in the U‐shaped structure between the posterior commissure and the third ventricle, corresponding to the SCO region. A strong positive signal was observed in the cytoplasm of SCO cells, particularly in the perinuclear region and in the apical surface of the SCO region in contact with the third ventricle. Pax6 expression was observed in the nuclei of cells that also express SCO‐spondin (Figure 2). These results show that Pax6 expression is maintained in the SCO of the mouse brain.

### 3.2 The SCO expresses marker proteins of neural stem/progenitor cells

**FIGURE 3:**
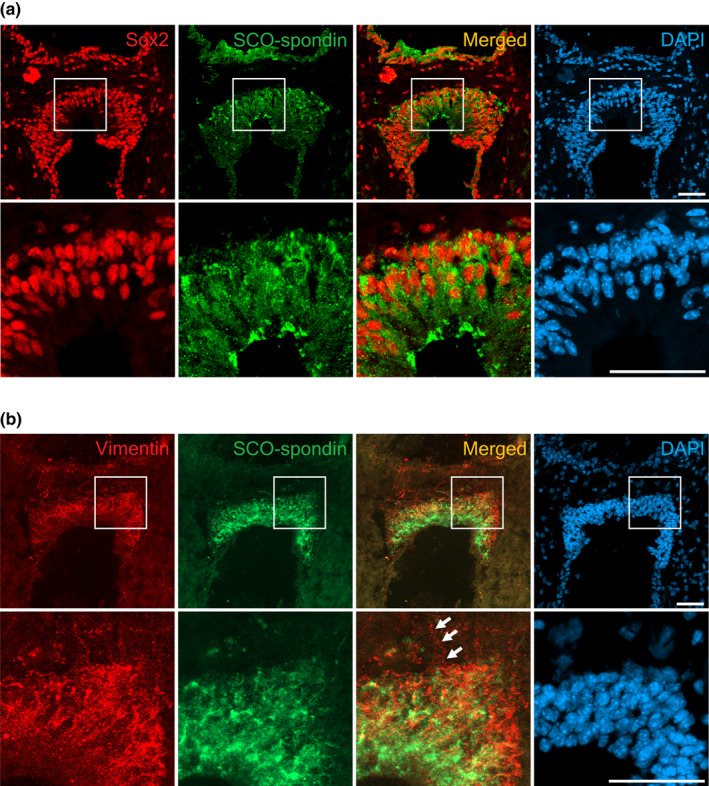
Expression of neural stem cell markers, i.e. Sox2 and vimentin, in the SCO region of the adult mouse brain. Sox2 immunostaining was performed on brains fixed with 4% PFA overnight and exposed to antigen retrieval solution by boiling in the citric acid solution for 12 min. Vimentin immunostaining was performed on brains fixed with 4% PFA for 5 min and no antigen retrieval. (a) Sox2 (red) is expressed in the nucleus of cells that express SCO‐spondin (green). (b) Immunofluorescence of vimentin (red) and SCO‐spondin (green) in the SCO. Overlap in vimentin and SCO‐spondin expression in the cytoplasm of SCO cells can be seen in the higher magnification image. Arrows point to possible basal process from the SCO region. Nuclei are counterstained with DAPI (blue). Merge images show red and green channels only. Scale bars: 50 μm.

Since strong Pax6 expression was observed in SCO cells, we then examined whether the SCO could be a neurogenesis niche containing NSPCs. The NSPCs at the ventricular zone during embryonic neurogenesis strongly express Pax6 (Gotz et al., 1998; Osumi, 2001), and Pax6 expression is weakly maintained in the NSPCs of the subgranular zone of the dentate gyrus and SVZ of the lateral ventricles at the adult stage (Osumi et al., 2008). We performed immunostaining using NSPC markers, i.e. Sox2 and vimentin. Sox2 is a transcription factor expressed in embryonic and adult NSPCs to maintain stemness properties (Ellis et al., 2004; Zappone et al., 2000), whilst vimentin is a cytoskeletal protein strongly expressed in NSPCs (Cochard & Paulin, 1984). Sox2 expression was observed in the nucleus of cells expressing SCO‐spondin (Figure 3a). On the contrary, the vimentin signal overlapped with the SCO‐spondin signal in the cytoplasm of SCO cells (Figure 3b). Vimentin expression was observed throughout the SCO region, with strong expression around the nucleus of SCO cells. Possible basal processes projecting from the SCO region to the posterior commissure were also observed (Figure 3b). Our results indicate that NSPC markers Pax6, Sox2, and vimentin are expressed in the SCO of the adult mouse brain, suggesting a possibility that the SCO might keep the potential for neurogenesis.

### 3.3 Proliferating cells in the SCO region

**FIGURE 4:**
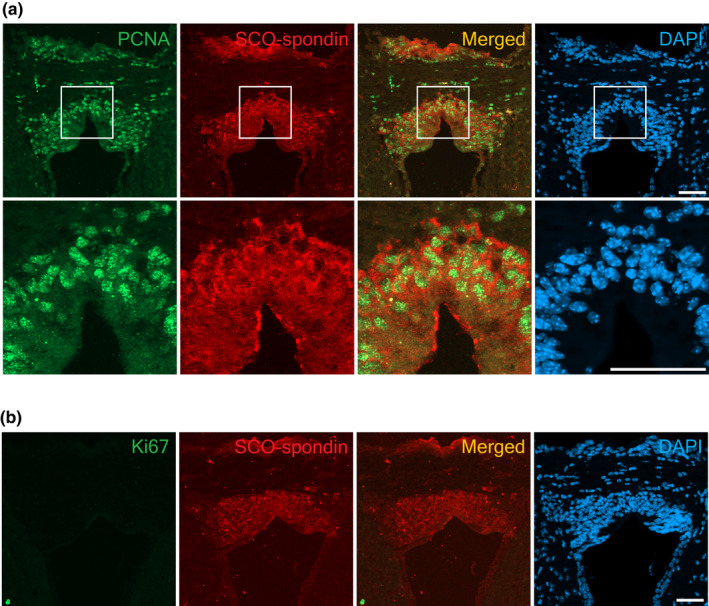
Expression of proliferating cell markers in the SCO region of the adult mouse brain fixed with 4% PFA overnight and exposed to antigen retrieval by boiling in the citric acid solution for 12 min. (a) PCNA (green) expression is observed in SCO‐spondin (red) expressing cells. (b) no Ki67 positive cells are in the SCO region. Nuclei are counterstained with DAPI (blue). Merge images show red and green channels only. Scale bars: 50 μm.

**FIGURE 5:**
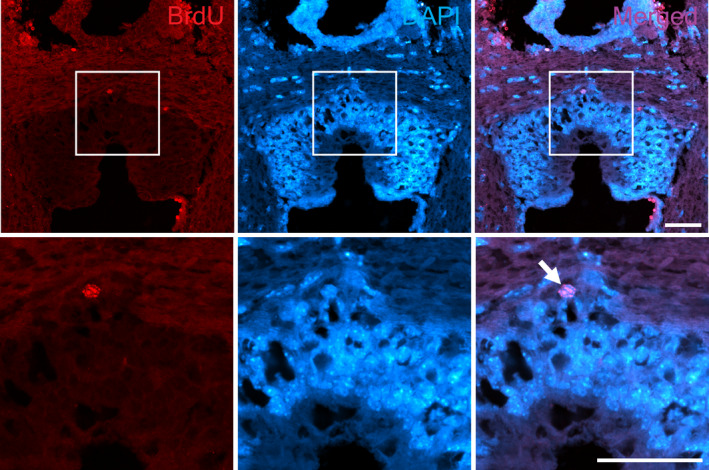
BrdU‐labelling in the SCO region of the adult mouse brain. P84 male mice received 50 mg/mL BrdU (P75 – P83) and coronal sections were examined for BrdU immunostaining with antigen retrieval using 2 M HCl at 37°C for 10 min. Only a few BrdU‐labelled cells are observed in the SCO region (arrow). Nuclei are counterstained with DAPI (blue). Scale bars: 50 μm.

Since NSPCs have proliferating activity, immunostaining was performed using antibodies against proliferating cell markers PCNA and Ki67 on sections of the SCO. PCNA is a protein complex that binds to DNA as part of the replication machinery in eukaryotes and is highly expressed during the late G1 and S phases of the cell cycle (Kurki et al., 1986; Takahashi & Caviness, 1993). Ki67 is present in proliferating cells at all phases of the cell cycle and is a useful proliferative marker during adult neurogenesis (Kee et al., 2002). In the three‐month‐old mouse brain, strong PCNA expression was observed in cells expressing SCO‐spondin (Figure 4a). Ki67, on the contrary, was undetectable in the SCO cells, whilst a few positive cells were detected in the dentate gyrus of the hippocampus, where proliferating NSPCs exist (Figures 4b and S1). Therefore, the SCO of the adult mouse contained proliferating cells expressing PCNA but not Ki67. To confirm cell proliferation, BrdU assays were performed. Only a few BrdU positive cells were observed at the periphery of the SCO, whilst several BrdU positive cells were observed in the subgranular zone of the hippocampus (Figures 5 and S1). SCO‐spondin signal was very weak or not observed in BrdU labelled sections possibly due to the harsh HCl treatment for BrdU antigen retrieval. These results suggest that the SCO cells may have a potential of proliferation yet be quiescent.

### 3.4 Other cell type in the SCO of adult mouse brain

**FIGURE 6:**
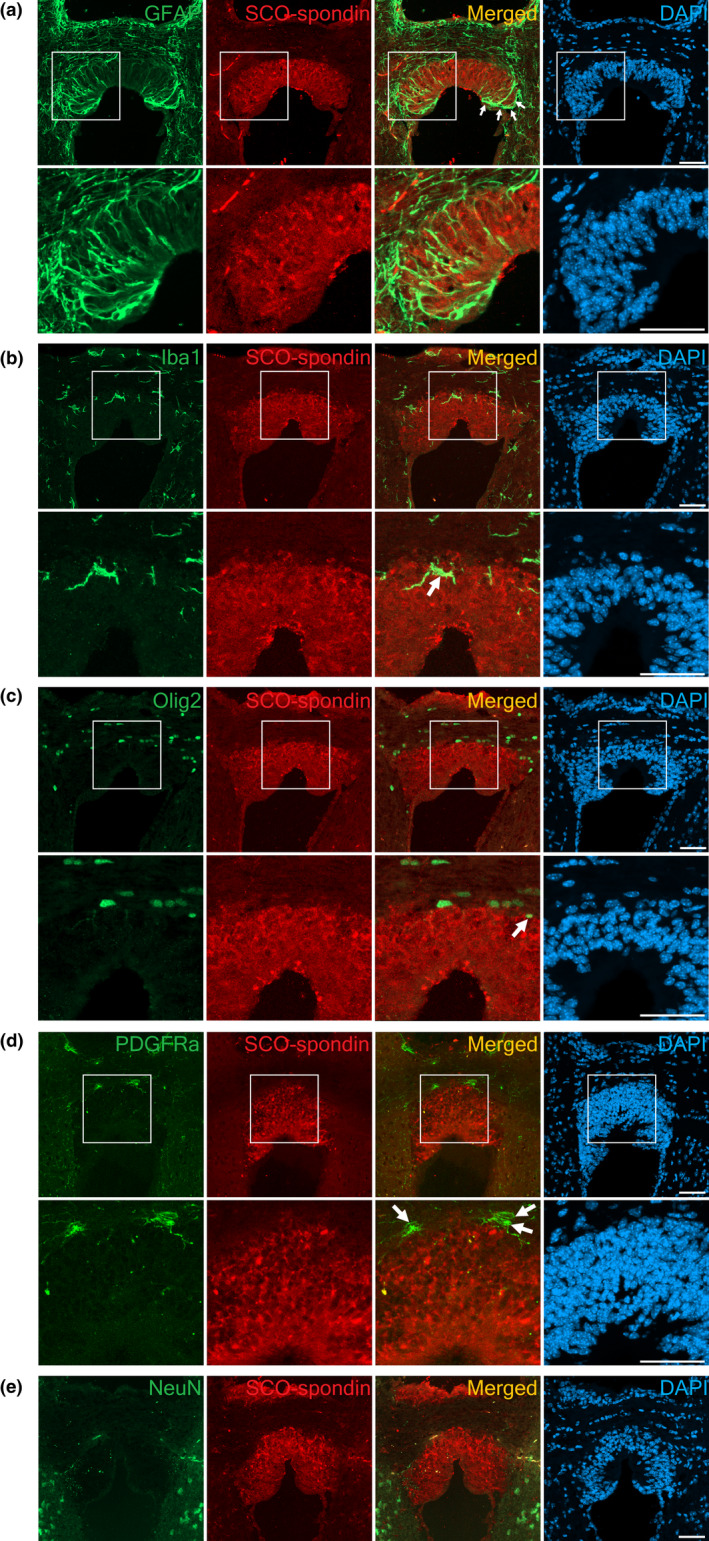
Expression of markers for various cell types in the SCO region of the adult mouse brain. GFAP, Iba1, Olig2 and NeuN immunostaining were performed on brains fixed with 4% PFA overnight and exposed to antigen retrieval solution by boiling in the citric acid solution for 12 min. PDGFRα immunostaining was performed on brains fixed with 4% PFA for 5 min. (a) Astrocyte marker GFAP is expressed in the SCO region, with the highest intensity in the lateral convexities (arrows). Note that ependymal cells also express GFAP. A few cells positive for (b) Iba1 for microglia, (c) Olig2 for oligodendrocytes and (d) PDGFRα for oligodendrocyte progenitor cells are only observed near/at the periphery of the SCO (arrows). Magnification of the area squared in the PDGFRα (red) and SCO‐spondin (green) double‐immunostaining shows that these signals do not overlap. (e) Brain cell type marker NeuN for neurons is undetectable in the SCO. Nuclei are counterstained with DAPI (blue). Merge images show red and green channels only. Scale bars: 50 μm.

To determine cell types in the SCO of adult mice, we used GFAP for astrocytes (Bignami et al., 1972), Iba1 for microglia (Imai et al., 1996; Ito et al., 1998), NeuN for neurons (Mullen et al., 1992), Olig2 for oligodendrocytes (Ligon et al., 2004) and PDGFRα for oligodendrocyte progenitor cells (Hall et al., 1996; Matsumoto et al., 2011) together with SCO‐specific marker SCO‐spondin. Only GFAP was expressed within the SCO region (Figure 6a). Although GFAP is a commonly used marker for astrocytes, it is also reported to be expressed in ependymal cells of the SCO (Redecker, 1989) as well as in NSPCs of the adult brain (Doetsch et al., 1999; Seri et al., 2001). GFAP expression was observed throughout the SCO region. Faint GFAP signals were visible in the middle part of the SCO region whilst strong GFAP expression was observed in the lateral parts (Figure 6a). This observation is consistent with the prominent GFAP immunoreactivity in the lateral convexities reported in the SCO region of the Mongolian gerbil (Redecker, 1989). A few Iba1‐positive cells, morphologically identified as microglia, were also observed in the SCO and surrounding regions (Figure 6b). A small number of Olig2 positive cells were observed in the periphery of the SCO, close to the posterior commissure (Figure 6c). On the other hand, cells expressing PDGFRα were observed along the edges of the SCO (Figure 6d). We did not detect any expression of NeuN within the SCO region (Figure 6e). Our results suggest that the SCO was formed by a uniform type of cells, showing features similar to neuroepithelial cells or NSPCs.

## 4 DISCUSSION

In this study, we revealed that the Pax6 expression was maintained in the SCO region at the postnatal stages from neonatal to adult. Pax6 was co‐expressed with SCO‐spondin, a specific marker of the SCO region. We also showed that the SCO cells expressed NSPC markers Sox2 and vimentin as well as a proliferation marker PCNA. However, the expression of Ki67, another proliferation marker, and incorporation of BrdU, were undetectable in the SCO, suggesting that the SCO cells could be quiescent regarding cell division. GFAP, a marker of astrocytes as well as NSPCs, and Olig2, a marker of oligodendrocytes, were also expressed in the SCO. The expression of markers for other cell types, such as neurons and oligodendrocyte progenitor cells, was absent within the SCO.

At this point, we could not conclude whether the SCO is a neurogenesis niche or not, even though the SCO cells have a similar feature to the neuroepithelial cells or NSPCs. The neurogenic potential of the SCO region has also been suggested by the study by Vera et al. (2013). In their study, in vivo loss of SCO‐spondin, a glycoprotein that is specifically produced and secreted by SCO cells showed an increase in proliferation of neuroepithelial cells as well as a decrease in neurodifferentiation in the diencephalon and mesencephalon during chick brain development. This suggests that SCO‐spondin is an important regulator of proliferation and differentiation of neuroepithelial cells. In the case of mouse early brain development, it is shown that neuroepithelial cells expressing Pax6 differentiate into the secretory SCO cells, and Pax6 expression is essential for the SCO differentiation (Estivill‐Torrus et al., 2001). Persistent expression of Pax6 in the SCO at postnatal stages may suggest a necessity of the transcription factor for not only SCO differentiation but also for its functional maintenance.

The SCO is a part of the circumventricular organs (CVOs) together with the organum vasculosum of the lamina terminalis (OVLT), subfornical organ (SFO), median eminence (ME), pineal gland (PG), and area postrema (AP). The CVOs have been gaining popularity as NSC niches, as demonstrated by the following studies. Some CVOs in adult rodents have been shown to express markers for NSCs such as nestin, vimentin and Sox2 (Bennett et al., 2009; Lee et al., 2012) as well as for neural progenitor cells such as Math1 and Mash1 (Hourai & Miyata, 2013). CVOs also show abilities for proliferation and generation of neurons or glial cells (Bennett et al., 2009; Hourai & Miyata, 2013; Lee et al., 2012). Bennett et al. (2009) have also shown that nestin and GFAP are expressed in the SCO region of the adult rat brain. SCO cells also express other neuroepithelial cell markers Nestin (Lendahl et al., 1990), Notch 1 (Lutolf et al., 2002), Hes1 and Hes3 (Hatakeyama et al., 2004; Sasai et al., 1992), Occludin (AakuSaraste et al., 1996), E‐cadherin (Chen et al., 2015; Karpowicz et al., 2009), MSI‐1 (Sakakibara et al., 1996), BMI‐1 (Molofsky et al., 2003) at the RNA level based on Allen Brain Atlas (Figure S2). These similarities between the SCO and the CVOs and their common features of cell types further support the possibility that the SCO could be a potential NSPC niche.

We also reported in this study that the SCO region in the adult mice brain expressed the NSPC markers Sox2 and vimentin, and a proliferation marker PCNA, and suggest that SCO cells could be potential NSPCs in the adult mouse brain. It should be noted, however, that neither Ki67 expression (strong at G_2_/M phase) nor BrdU incorporation (S phase) was observed in the SCO region, implying that SCO cells might be arrested at the G_1_ phase. Therefore, the SCO cells might be quiescent in cell division. Recently, it has been reported that tanycyte‐like ependymal cells in the CVOs and the central canal (CC) show a neural stem cell‐like phenotype in the adult mouse brain (Furube et al., 2020). In their study, tamoxifen‐induced EGFP labelling under the control of Nestin‐CreERT2 transgene has identified NSPCs and reported the distribution of EGFP‐labelled ependymal cells in the OVLT, SFO, the central canal and the arcuate nucleus (Arc) of the hypothalamus but not in the SCO. The EGFP‐labelled tanycyte‐like ependymal cells of the OVLT and SFO express both GFAP and Sox2 but not Pax6, whilst the cells of the CC expressed those three marker proteins (Furube et al., 2020), which is similar to our results in the SCO. Furube et al. (2020) also observed a few BrdU‐labelled tanycytes/tanycyte‐like ependymal cells in the OVLT, SFO, Arc and CC, with the number of BrdU positive cells significantly increasing by infusion of FGF‐2 and EGF into the lateral ventricle. We only observed a few BrdU positive cells in the SCO, which could be due to the absence of growth factor stimulation. The proliferation of SCO cells may also require stimulation by suitable growth factors such as FGF‐2 and EGF. Taken together, similarities in NSPC markers and BrdU incorporation between tanycytes/tanycyte‐like ependymal cells in CVOs and SCO cells further highlight the potential of the SCO cells as NSPC.

It has been reported that the SCO consists of ependymal and hypendymal cells in different layers (Rodriguez et al., 1984; Schoebitz et al., 1986; Sterba et al., 1982). Ependymal cells of the SCO have a bipolar morphology (Redecker, 1989; Rodriguez et al., 1984; Viehweg & Naumann, 1996), resembling radial glia and tanycytes. Ependymal cells have been defined as tall cylindrical secretory cells with the nucleus in the basal position that line the ventricular cavity, forming the ependymal layer of the SCO (Rodriguez et al., 1992; Rodriguez et al., 1998). The hypendymal cells of the SCO, in contrast, are also bipolar cells but with a thin apical pole and basal process (Viehweg & Naumann, 1996). Hypendymal cells are secretory cells between the ependymal layer and the posterior commissure, forming the hypendmal layer of the SCO (Rodriguez et al., 1992; Rodriguez et al., 1998). This definition has been used to differentiate ependymal and hypendymal cells in the SCO of adult rat (Viehweg & Naumann, 1996), chicken and duck (Schoebitz et al., 1986) and bovine (Montecinos et al., 2005). However, it was difficult to distinguish ependymal and hypendymal cells in the adult mouse SCO coronal sections following these definitions since the SCO‐spondin staining pattern of SCO cells in the adult mouse was uniform from the basal region where the nuclei are located, up to the apical region in contact with the third ventricle. Some cells even show strong SCO‐spondin in the perinuclear region (Figures 2 and S3a). We have analysed the adult rat SCO and observed that the basal region exhibited weak SCO‐spondin expression whilst the apical region showed strong SCO‐spondin expression thus, forming a distinct nuclear region for the ependymal cells (Figure S3a). The rat SCO immunostaining allowed easier identification of a few secretory cells in between the ependymal layer and the posterior commissure, which could be potential hypendymal cells (Figure S3a). This SCO‐staining pattern is significantly different in the two rodent species, suggesting the adult mouse hypendymal layer may not be developed as in adult rat SCO. Immunostaining of the sagittal section of the adult mouse SCO with SCO‐spondin also helped to distinguish ependymal and hypendymal cells and identified very few potential hypendymal cells between the ependymal cells and the posterior commissure along the rostro‐caudal length of the SCO (Figure S4). This is consistent with the description of the adult mouse SCO having few hypendymal cells by Rodriguez et al. (1992). Almost all the cells in the SCO region between the posterior commissure and the third ventricle of the adult mouse were Pax6 and Sox2 positive, at 90% and 100%, respectively. The few hypendymal cells observed above the ependymal layer using the sagittal section of the adult mouse brain were also positive for Pax6 and Sox2 (Figure S4a,b). These results suggest that both ependymal and hypendymal cells of the SCO show features of neuroepithelial cells, and not only a subpopulation of ependymal cells. Immunostaining of coronal sections of adult rat SCO also showed that both ependymal and hypendymal cells were Pax6 and Sox2 positive (Figure S3b,c), supporting our results in the adult mouse SCO.

In summary, our data demonstrated that the SCO cells have neuroepithelial cell characteristics, suggesting that the SCO is a possible adult neural stem cell niche. Hence, further study is needed to understand this unique brain region. Expanding our knowledge on the SCO focusing on Pax6 would be beneficial to understand congenital hydrocephalus because clinical cases of hydrocephalus have been reported in WAGR syndrome, in which *PAX6* is one of the causative genes (Demir et al., 2011; Jung et al., 2006).

## AUTHOR CONTRIBUTIONS

H.I. and N.O. designed the project. L.G.C., H.I., K.H., S.A., S.Y. and T.K. performed experiments. L.G.C., H.I., and N.O. wrote the paper. All authors approved the article.

## Supporting information


Figure S1

Figure S2

Figure S3

Figure S4

## Data Availability

Data are available on request from the authors.
