# Supplementary material for: The subcommissural organ maintains features of neuroepithelial cells in the adult mouse
Source: J Anat. 2022 May 31;241(3):820–30. doi: 10.1111/joa.13709 (PMC9358730; doi:10.1111/joa.13709)
Supplement: Supplementary file 1 — Figure S1 [file JOA-241-820-s001.pdf]

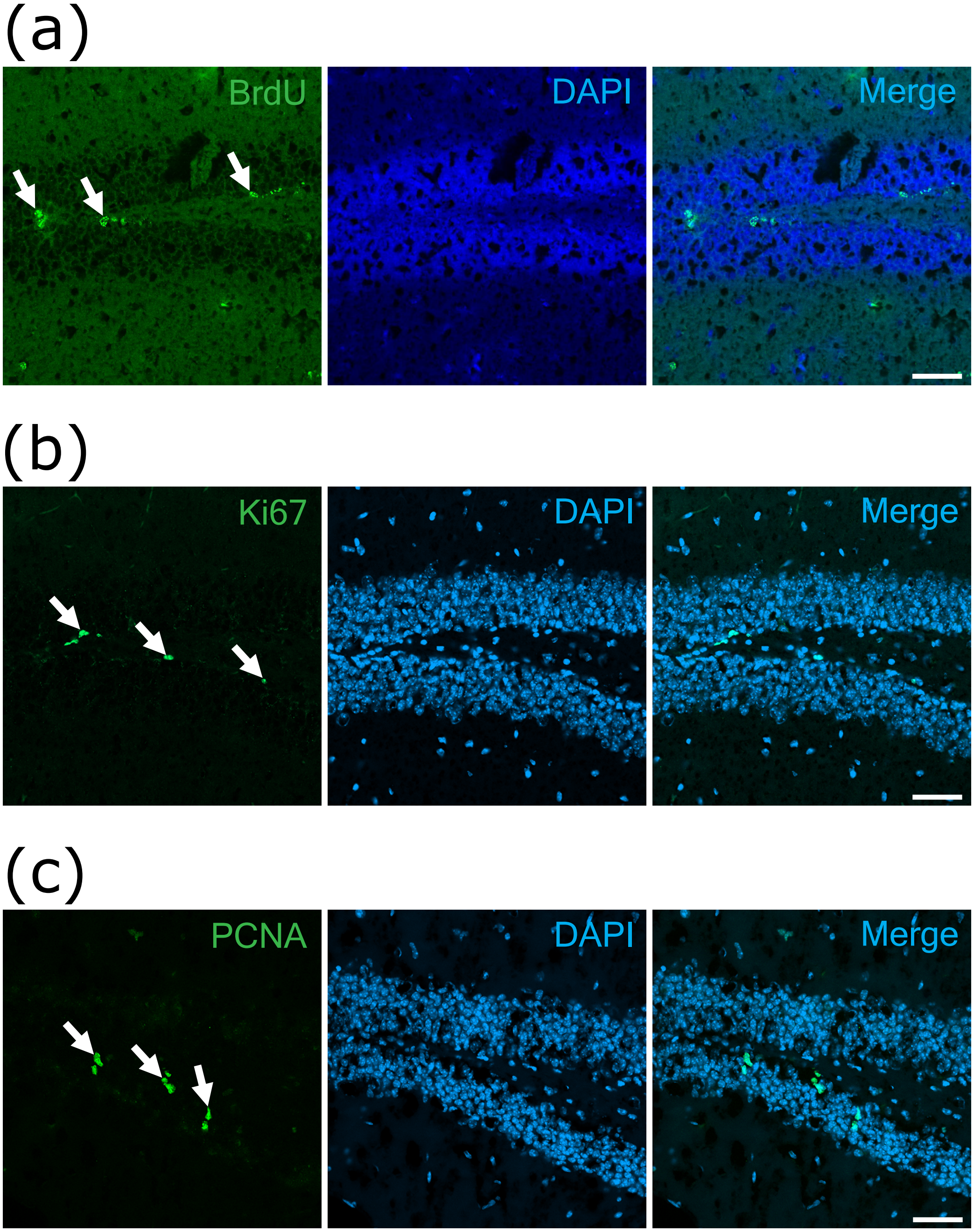


**Supplemental Figure S1.** Expression of proliferating cell markers in the hippocampus of the adult mouse brain. (a) BrdU-labelled, (b) Ki67 positive, and (c) PCNA positive cells (arrows) are detected in the hippocampus of the adult mouse brain. Nuclei are counterstained with DAPI (blue). Scale bars: 50 μm.
