# Supplementary material for: The subcommissural organ maintains features of neuroepithelial cells in the adult mouse
Source: J Anat. 2022 May 31;241(3):820–30. doi: 10.1111/joa.13709 (PMC9358730; doi:10.1111/joa.13709)
Supplement: Supplementary file 2 — Figure S2 [file JOA-241-820-s002.pdf]

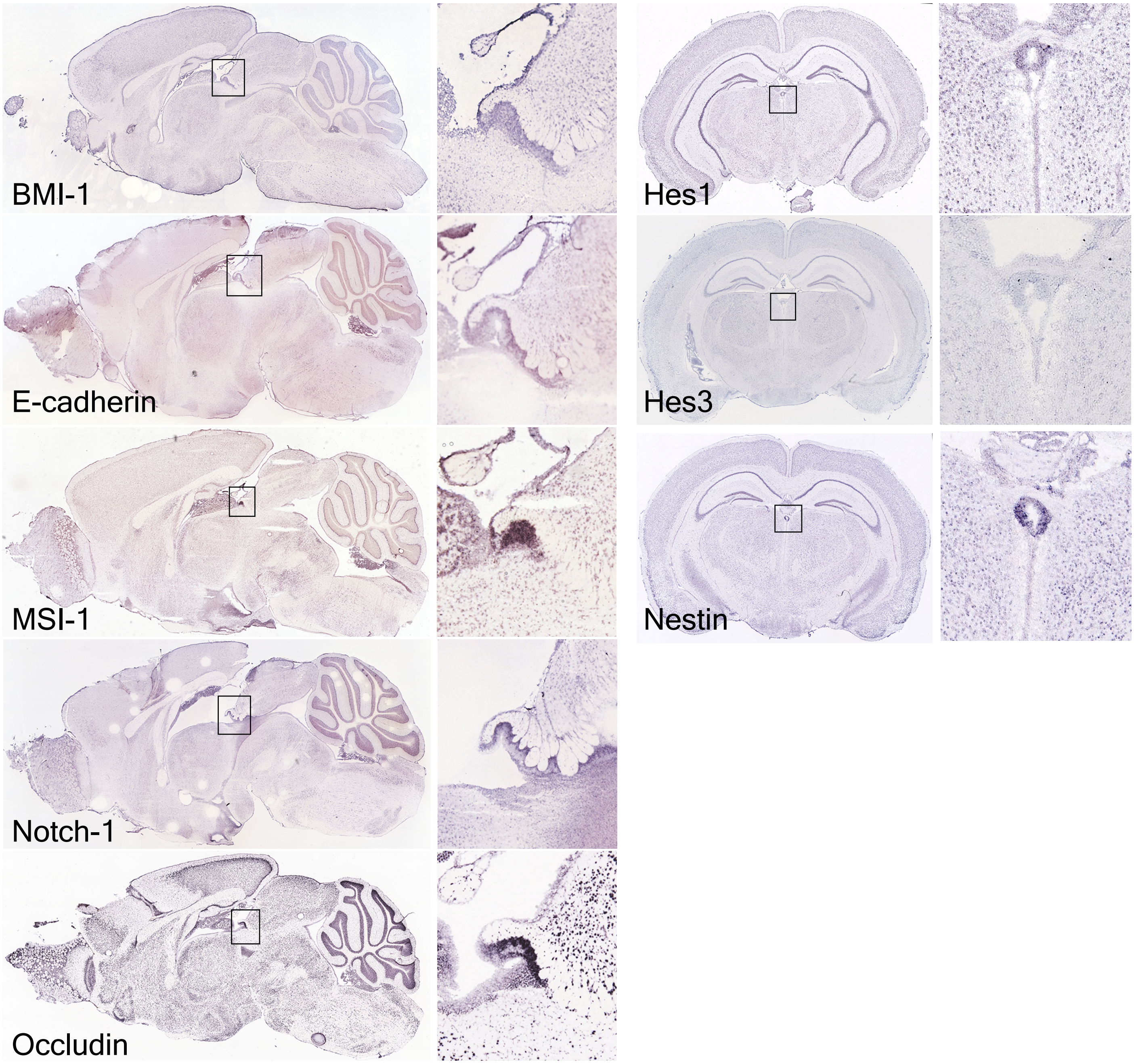

Supplemental Figure S2. Expression of other neuroepithelial cell markers in the SCO region of the adult mouse brain. In situ hybridization data from the Allen Mouse Brain Atlas shows expression of neuroepithelial cell markers BMI-1 (http://mouse.brain-map.org/gene/show/11937), E-cadherin (http://mouse.brain-map.org/experiment/show/70918862), Hes1 (http://mouse.brain-map.org/experiment/show/556), Hes3 (http://mouse.brain-map.org/experiment/show/2287), Nestin (http://mouse.brain-map.org/experiment/show/1387), Notch-1 (http://mouse.brain-map.org/experiment/show/70593326), MSI-1 (http://mouse.brain-map.org/experiment/show/74509595), and Occludin (http://mouse.brain-map.org/experiment/show/79394328) in coronal and sagittal sections of P56 mice (Lein et al., 2007; Allen Institute for Brain Science, 2004).

REFERENCES:

Allen Institute for Brain Science (2004) Allen Mouse Brain Atlas [dataset]. Available from http://mouse.brain-map.org

Lein, E.S., Hawrylycz M.J., Ao N., Ayres M., Bensinger A., Bernard A., et al. (2007) Genome-wide atlas of gene expression in the adult mouse brain. Nature, 445(7124), 168-176. https://doi.org/10.1038/nature05453
