# Supplementary material for: The subcommissural organ maintains features of neuroepithelial cells in the adult mouse
Source: J Anat. 2022 May 31;241(3):820–30. doi: 10.1111/joa.13709 (PMC9358730; doi:10.1111/joa.13709)
Supplement: Supplementary file 3 — Figure S3 [file JOA-241-820-s003.pdf]

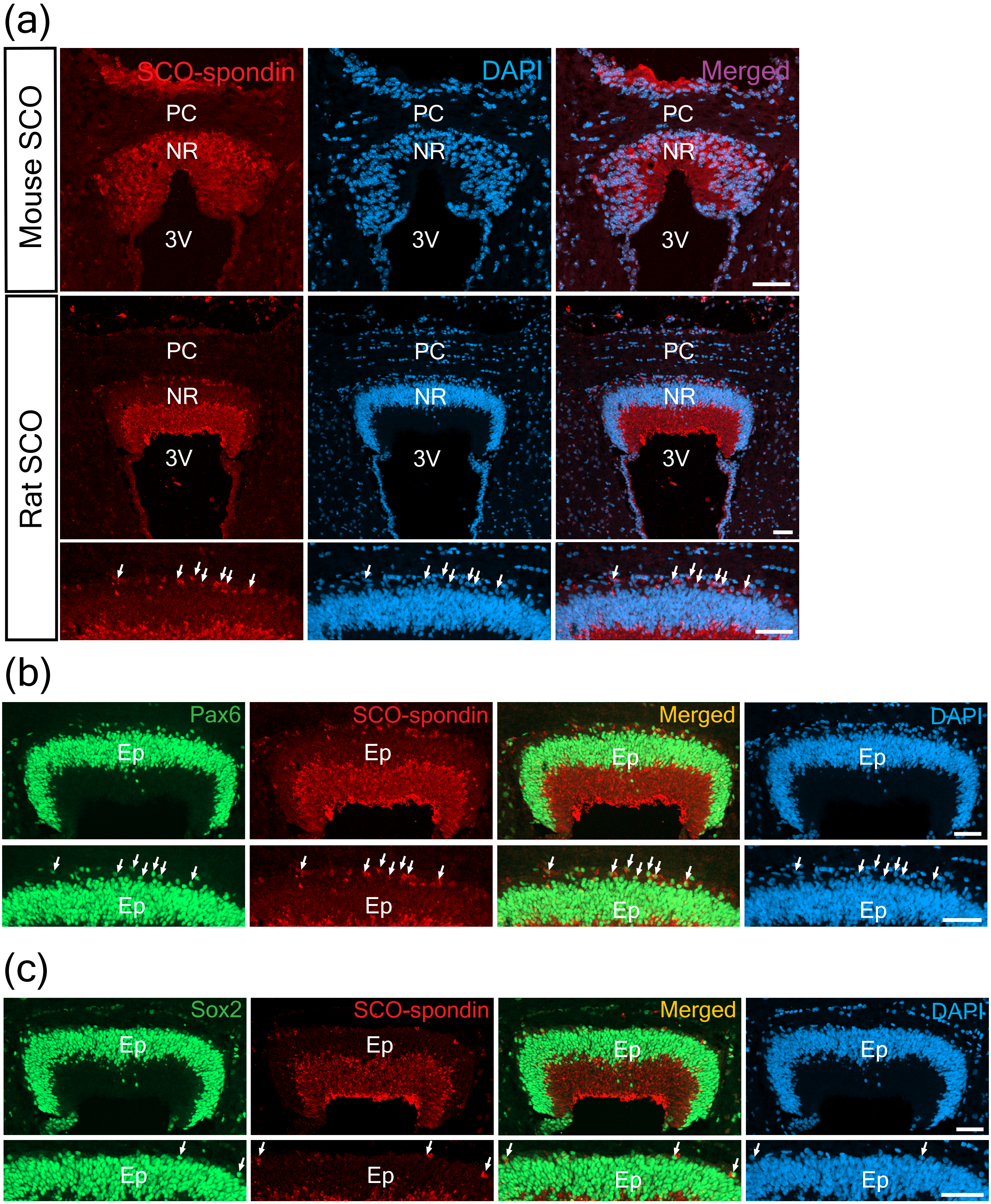

Supplemental figure 3. Immunostaining in the SCO region of the adult rat brain fixed with 4% PFA overnight and exposed to antigen retrieval solution by boiling in citric acid solution for 12 minutes (a) SCO-spondin staining pattern in the adult rat SCO shows weak staining pattern in the nuclear region compared to the adult mouse SCO allowing better visualization of hypendymal cells between the ependymal layer and the posterior commissure. (b) Pax6 and SCO-spondin double staining was performed using rabbit anti-Pax6 and goat anti-SCO-spondin antibodies as primary antibodies. Hypendymal cells expressing SCO-spondin (red) are also Pax6 positive (green). (c) Sox2 and SCO-spondin double staining was performed using goat anti-Sox2 and rabbit anti-SCO-spondin antibodies as primary antibodies. Hypendymal cells between the ependymal layer and the posterior commissure are SCO-spondin (red) positive and Sox2 (green) positive. Nuclei are counterstained with DAPI (blue). Merge images show red and green channels only. 3V: third ventricle, Ep: ependymal cells, NR: nuclear region, PC: posterior commissure. Arrows indicate hypendymal cells. Scale bars: 50 µm.
