# Supplementary material for: The subcommissural organ maintains features of neuroepithelial cells in the adult mouse
Source: J Anat. 2022 May 31;241(3):820–30. doi: 10.1111/joa.13709 (PMC9358730; doi:10.1111/joa.13709)
Supplement: Supplementary file 4 — Figure S4 [file JOA-241-820-s004.pdf]

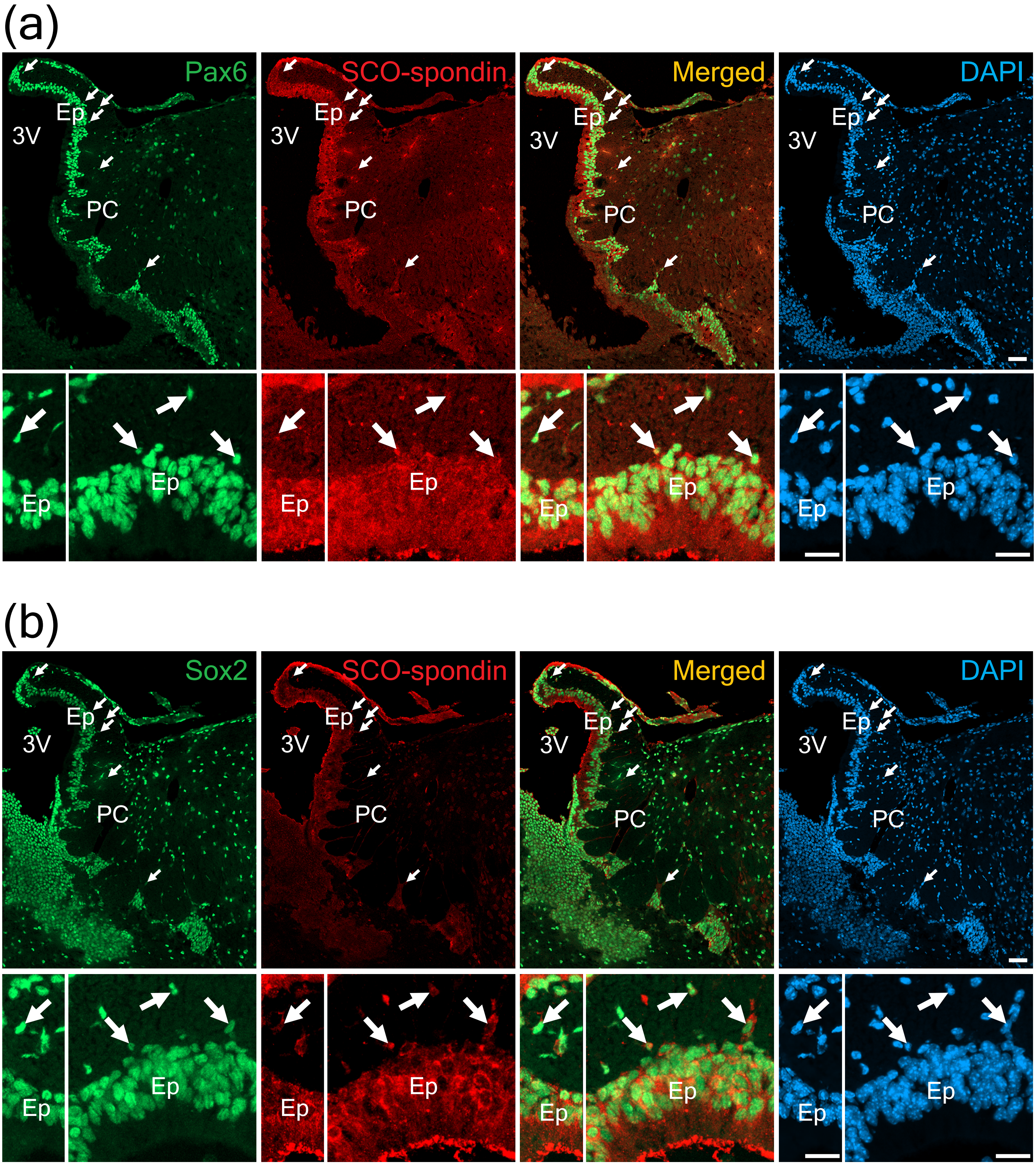

Supplemental Figure S4. Expression of neural stem cell markers, i.e., Pax6 and Sox2, in consecutive sagittal sections of the SCO region of the adult mouse brain. (a) Pax6 and SCO-spondin double staining was performed on brains fixed with 4% PFA overnight and exposed to antigen retrieval solution by boiling in citric acid solution for 12 minutes. Rabbit anti-Pax6 and goat anti-SCO-spondin antibodies were used as primary antibodies. Very few Pax6 (green) and SCO-spondin (red) positive cells are observed above the ependymal cells (b) Sox2 and SCO-spondin double staining was performed on brains fixed with 4% PFA overnight and exposed to antigen retrieval solution by boiling in citric acid solution for 12 minutes. Goat anti-Sox2 and rabbit anti-SCO-spondin antibodies were used as primary antibodies. Few cells positive for Sox2 (green) and SCO-spondin (red) are observed above the ependymal layer. Nuclei are counterstained with DAPI (blue). Merge images show red and green channels only. 3V: third ventricle, Ep: ependymal cells, PC: posterior commissure. Arrows indicate hypendymal cells. Scale bars: 50 μm. Higher magnification image scale bars: 20 μm.
